# To Burn or Not to Burn? Effect of Management Strategy on North American Prairie Vegetation for Public Urban Areas in Germany

**DOI:** 10.1371/journal.pone.0108588

**Published:** 2014-10-06

**Authors:** Anja Schmithals, Norbert Kühn

**Affiliations:** Department of Landscape Architecture and Environmental Planning, Chair of Vegetation Technology and Planting Design, Berlin Institute of Technology, Berlin, Germany; California State University, Fresno, CA, United States of America

## Abstract

North American prairie vegetation has been a role model for designing highly attractive plantings for German urban green spaces for the past decade. In combination with gravel mulch top layers on planting sites and non-selective maintenance techniques like mowing or burning, prairie plantings are considered to be cost-effective and low-maintenance. This study was undertaken to assess the impact of different maintenance strategies and especially the necessity of fire management on the development success of ornamental prairie plantings in central Europe. A four factorial split-plot-block design was set up for investigation of different mixtures of prairie species under varying management conditions (mow-only, mowing plus selective weeding, mowing plus weeding and burning) on two differing soil types (in-situ topsoil and in-situ topsoil with a graywacke gravel mulch top layer) over three years. Significant effects of maintenance strategy on mortality rates and vitality were documented for a number of target species, which responded species specifically, either being slightly affected by the burning or thriving on it. Those effects were mostly restricted to topsoil sites. A strong impact on weed species presence and abundance and resulting maintenance times was found on both soil types. On topsoil sites, mow-only treatment resulted in a short-term loss of the original planting due to extensive weed growth. Corresponding gravel mulch sites were generally less colonised and visually dominated by weeds. Differences between weeded and weeded plus burned sites were minor. Unexpectedly, weed species populations were mostly unaffected by the additional burning treatment, while maintenance times and costs increased. No overall benefit of fire management for the establishment of prairie plantings was documented. The most effective management combination proved to be mowing plus regular selective weeding measures on gravel mulched planting sites.

## Introduction

For the past few decades in Germany, structurally diverse and sophisticated plantings- particularly herbaceous plantings- have been declining in popularity, due to high maintenance costs and the lack of personnel with the relevant expertise [1], [2]. Inspired by the knowledge gained from ecological analysis of natural plant communities [3], [4], a new, more naturalistic approach to planting design was developed and is supported by an increased awareness of the beauty of native plant communities like meadows, calcareous grasslands or woodland edges [5]. To correspond with this “New German Style” [6], new maintenance concepts have been developed [7], [8] and economized by a high degree of mechanization [9].

The North American prairie first became a new model for German planting design concepts around the year 2000. The prairie idea was widely promoted in German landscape architecture and garden design magazines [10]–[17]. In addition to its great abundance of attractive species, the prairie ecosystem itself inspired a new approach to planting designs. A prairie planting was believed to be a self-sustainable, highly attractive plant community that could be maintained with very little effort [18]–[21]. In subsequent years, numerous ornamental prairie projects were initiated in public green spaces by parks departments (e. g. [22]–[25]), but none were monitored systematically to demonstrate whether the expected community stability and associated low maintenance requirement actually prevailed.

In natural prairie communities, disturbance on a regular basis is known to be a necessity for maintenance [26]–[28], with fire being a particularly high impact disturbance [29]. When burning is omitted from management, a loss of species with low growth form and small seeds, in particular legumes, results [30]. Prairie species are fire-adapted [31] and compensate for aboveground loss of biomass with their extensive root systems which allow them to regenerate successfully [32].

In the management of German public green spaces, prescribed burning could allow for effective control of native wintergreen weedy vegetation, while leaving the target species mostly unharmed. To date it remains unknown whether German prairie plantings can in fact be managed as extensively as believed and if fire is a mandatory disturbance. Reliable data on the actual care requirements are needed in terms of both time required for maintenance and appropriate techniques. Additionally, for prairie plantings to be a suitable alternative for German low-maintenance public planting designs, the prairie species in question must not only establish well, but also must be long-lived, vigorous and successful at reproducing in order to create visually attractive and truly sustainable plantings. In the face of climate and growing conditions that differ from their native habitats, these characteristics should not be taken for granted. None of these aspects have been empirically investigated before.

The effect of fire on natural prairie communities depends highly on when burning occurs during the year and the intervals between fire events. Burning in spring decimates wintergreen species alien to the prairie ecosystem [33] and reduces species richness, favouring the warm-season grasses [34], [35]. Accumulated ground level biomass is removed, resulting in increased soil temperatures, optimum sunlight exposure, a temporary nutrient surplus [36], [37] and improved conditions for germination [38]. Vegetation development is commonly stimulated, and late-developing grass species benefit the most [39]. Burning during the summer promotes subordinate species, increases species diversity and weakens the competitiveness of the *Gramineae* species [40]–[42]. Maximum forb species diversity is achieved with a burning regime every two to three years [43]. Shorter intervals between fires promote grass development and abundance. Longer intervals induce a general species impoverishment with higher forb ratios [26], [44]. Selective weed control is important in newly established North American prairie plantings, which are usually not burned during the first growing season [45].

With the picture of a diverse planting rich in forb species in mind, it is expected that the effect on community development caused by a biennial summer burning would suit the long-term design goals best [42], [43]. Unfortunately, this would destroy the visual effect of the planting at the peak of seasonal development for the rest of the growing season, which would not be acceptable in the context of public green space. Alternatively, an early spring burning should affect native wintergreen species disproportionately, without conflicting with public interests, although it risks directing development away from flowering plants towards a grass-dominated prairie plant community.

Mowing of ornamental prairie plantings in the public green could be another suitable maintenance technique. As with fire management, the effect of mowing on plant communities depends greatly on the time and frequency of cutting. Mowing of natural prairie communities facilitates forb species [46]–[48]. Diversity is generally reduced because the remaining litter layer acts as an establishment barrier against germination [49], with the small-seeded grasses being more heavily affected than many of the forb species [50].

Luz [51] tested various mowing techniques for extensive ornamental herbaceous plantings and found mowing with a brush cutter, a rotary mower or a flail mower (provided that the planting lacks a gravel mulch layer) to be time-saving alternatives to traditional cutting-back. He determined the optimum time for mowing to be late winter before the shoots of geophytes emerge. The time saved over cutting flowers back manually significantly reduces maintenance costs [9].

Again, in the context of managing German public plantings, dormant-season mowing together with subsequent raking away of the voluminous clippings might be an adequate maintenance strategy for urban prairie plantings. In this case, Central European species would be expected to prevail without selective suppression via burning. It is of interest whether selective weeding might prove necessary for long-term successful maintenance, despite the promised self-sustainability of prairie plant communities.

Our objective in this study was the investigation of the effects of different management techniques (fire, mowing and selective weeding) on the establishment of a mixed-planting of prairie forbs and grasses in an urban context. We specifically focused on the effects of additional burning measures vs. weeding-only management on establishment success, on individual target species' vitality, on control of weedy species and resulting maintenance effort.

## Work Schedule and Methods

### Ethics statement

The field studies did not involve endangered or protected species.

### Experimental setup and preparation

The experiment site was located on property of the Berlin Institute of Technology in the south-western part of Berlin (52.455215°N 13.298584°E). The Chair of Vegetation Technology and Planting Design at the Department of Landscape Architecture and Environmental Planning is the assigned administrative and decision-making authority for the study site.

The site had been used for experimental vegetable gardening and was abandoned in the summer of 2008. After clearing, the site was rototilled 40 cm deep in late 2008. In 2009, *Sinapis alba* was sown as an intermediate crop. The site was cleared again at the end of summer 2009.

A four factorial split-plot-block design (A/(B+C)/D−Bl) was established (total size 43.20 m×22.60 m) in September 2009) as described by Thomas [52] (pp. 156–157).

The factors were as follows:

A. Substrate (in-situ topsoil, 7 cm greywacke chippings 5/11 on top of in-situ topsoil)

B. Species diversity (32, 16, 8 species)

C. Grass-to-forb ratio (80∶20, 50∶50, 20∶80)

D. Management type [mowing (m), m + selective weeding (s), m + s + burning in early spring (b)].

Nine species mixes (three different species diversities combined with three different grass-to-forb ratios) were established in a split block design (factor combination B+C; 3.60 m×4.80 m each). Each mixture plot was subdivided into three subplots (split plot; 3.60 m×1.60 m each) which were assigned to the different management types. This basic unit was planted on i) topsoil and ii) topsoil with a layer of greywacke chipped gravel mulch on top (factor A), giving the study design 2×3×3×3 individual factor combination units. The study factors “soil type” and “management” forbid a randomization for practical reasons; therefore it was established as a fully balanced, non-randomized design. Three replicates were established. Plots were marked by wooden pegs at the corners. A 100 cm geomembrane was installed around the experiment site to inhibit clonal immigration of surrounding turf grasses and to reduce seed input. The same was done between soil types to prevent the gravel mulch of the elevated area from moving into the topsoil area.

Seedling plants with miniature root-balls (ø 4 cm) were planted at a density of 25 plants/m^2^ in an 8×18 grid mixed planting. The planting was followed over three years.

The study design was developed to allow for many factors to be considered economically. Substrate effects on management issues are the focus of this article. Research into the effects of the other factors, grass-to-forb ratio and species diversity, is ongoing and will be presented in the future.

### Species choice

Species were selected on the basis of suitability for dry-mesic to mesic soils, medium size and growth, attractiveness of flowers or seed heads, wild-growing origin and known or assumed horticultural potential. Transient forb species were included for the initial effect of the plantings, slow developers for long-term aspects. Altogether, three C4 grasses and 29 forb species were chosen.

Two nurseries carried out the cultivation according to their species expertise and logistical capacities. Twenty-five of the target species were planted in 2009. The remainder of the delivered species were either of insufficient quality or quantity and needed to be substituted. Additional cultivation was started in early 2010. Those seedlings were planted in September 2010. See table 1 for final species choice and species mixture compositions.

**Table 1:** Complete list of study species and mixture compositions.

Mixture	1	2	3	4	5	6	7	8	9
# of species in the mixture	32	32	32	16	16	16	8	8	8
Grass-to-forb ratio	80∶20	50∶50	20∶80	80∶20	50∶50	20∶80	80∶20	50∶50	20∶80
Species	Quantity in the mix								
*Agastache foeniculum*	1	2	3						
*Amorpha canescens*	1	2	2	1	1	2			
*Asclepias tuberosa*	1	2	6	2	6	10	7	15	29
*Asclepias verticillata*	1	3	4						
*Aster oblongifolius*	1	3	5	2	6	9			
*Baptisia australis*	1	1	2	1	1	2	1	2	3
*Bouteloua curtipendula*	38	24	9	38	24	9	38	24	9
*Dalea purpurea*	1	2	1						
*Echinacea angustifolia*	1	4	7	4	9	14	9	24	36
*Echinacea pallida*	1	3	4						
*Echinacea paradoxa*	1	2	4						
*Eryngium yuccifolium*	1	3	5						
*Liatris aspera*	1	3	4						
*Liatris ligulistylis*	1	4	6	4	8	12			
*Monarda bradburiana*	1	2	3						
*Oenothera macrocarpa*	1	2	4	3	7	11			
*Oligoneuron album*	1	2	3	2	6	9	7	18	30
*Parthenium integrifolium*	1	2	4						
*Penstemon grandiflorus*	1	2	3						
*Penstemon hirsutus*	1	3	4	2	8	12			
*Penstemon ovatus*	1	3	4						
*Pycnanthemum pilosum*	1	3	4						
*Pycnanthemum tenuifolium*	1	3	5						
*Ratibida columnifera var. pulcherrima*	1	2	4						
*Rudbeckia hirta*	1	2	4						
*Rudbeckia missouriensis*	1	2	5	2	7	11			
*Rudbeckia triloba*	1	3	4	2	4	6	5	13	17
*Schizachyrium scoparium*	38	24	10	38	24	10	38	24	10
*Solidago missouriensis*	1	2	3	2	4	7			
*Sporobolus heterolepis*	39	24	10	39	24	10	39	24	10
*Verbena hastata*	1	2	4	2	5	10			
*Verbena stricta*	1	3	4						

### Study site maintenance

Each year in late winter when the soil was still frozen and the weather was dry (usually mid-February), the site was mowed to a height of 10 cm with a sickle bar mower attached to a walking-tractor (cutting width 120 cm). The clippings were removed by raking. Burning was usually undertaken in mid-March, when native vegetation came into leaf. A three-wheeled mobile weed burner (Ecoflame Select 500) fuelled by a propane gas cylinder with an effective burning width of 50 cm and a gas consumption rate of 3 kg/h was used to burn the plots until standing vegetation and surface litter were carbonised.

Selective weeding was undertaken monthly between April and September. Weeds growing above the general plant canopy and weeds that were visually disturbing were pulled by hand and removed from the plots. All woody vegetation (mainly *Betula pendula*, *Acer platanoides, Salix* spp.) and certain herbaceous species (*Cirsium arvense, Humulus lupulus, Solidago canadensis*) were consistently removed regardless of their size using weeding tools if necessary.

Weeds that grew outside of the selective weeding plots (s- and b-treatments) were allowed to develop. No further maintenance was undertaken.

### Data collection

The time required for maintenance treatments was recorded plot-wise, except for mowing and raking, which were measured at the level of substrate units.

Survival of planted individuals was recorded in early June and September for three years starting in 2011, as were cover values of grasses, forbs, and bare ground. Identity and cover of weedy species were determined for all plots in June and September prior to selective weeding measures. Vitality criteria (e. g. basal diameter, number of generative shoots) were assessed in July and September for selected species based on the seasonal development of the traits to be recorded. For each species to be assessed, two different vitality criteria were determined. Assessment of vitality aspects was only undertaken within the 32-species plots, where all of the species to be assessed were present. Sample size per species was capped at five individuals per study plot. For all of the grass species and *Echinacea angustifolia*, this required a permanent marking of the selected individuals from a generally greater stock. For the remaining species, this included all planted individuals.

### Limitations of the study

Planting material of some species was partly of insufficient quality (*Echinacea angustifolia*, *Oligoneuron album*), which may have accounted for high mortality rates during the establishment phase.

Because study site establishment occurred over two years, it was difficult to compare first year mortality rates, since establishment conditions differed greatly between years (two weeks of unfavourable weather after planting in 2009; safe-site effects for seedling establishment due to standing vegetation in 2010 accompanied by moderate temperatures). The species used in this study were a non-representative selection of the natural diversity found in native prairie plant communities. Results found within this study should not be applied to German urban ornamental prairie plantings in general but are most likely species specific.

### Statistical analysis

Statistical tests were undertaken using R 3.0.2 [53].

Where mortality data, total coverage values and vitality data of target species were not sufficiently normal for parametric analysis, they were either successfully logit-transformed or non-parametric methods were used (Kruskal-Wallis test in lieu of ANOVA). Count data of numbers of weedy species were analysed via generalised linear mixed modelling with a Poisson error distribution. Coverage values of weedy species were analysed via generalised linear modelling with either a Gamma or a Tweedie error distribution. Similarity analysis of weed species communities was done by permutational multivariate ANOVA using distance matrices (Bray-Curtis indexes for abundance data and Jaccard indexes for presence/absence data). Management duration data were analysed via Wilcoxon-Mann-Whitney tests.

## Results

### Effect of maintenance type on target species establishment

At the level of species mixture, no significant effect of maintenance type on mortality rates was found between June 2010 and June 2013, either for the whole of the study layout or at the level of substrate units.

**Figure 1:**
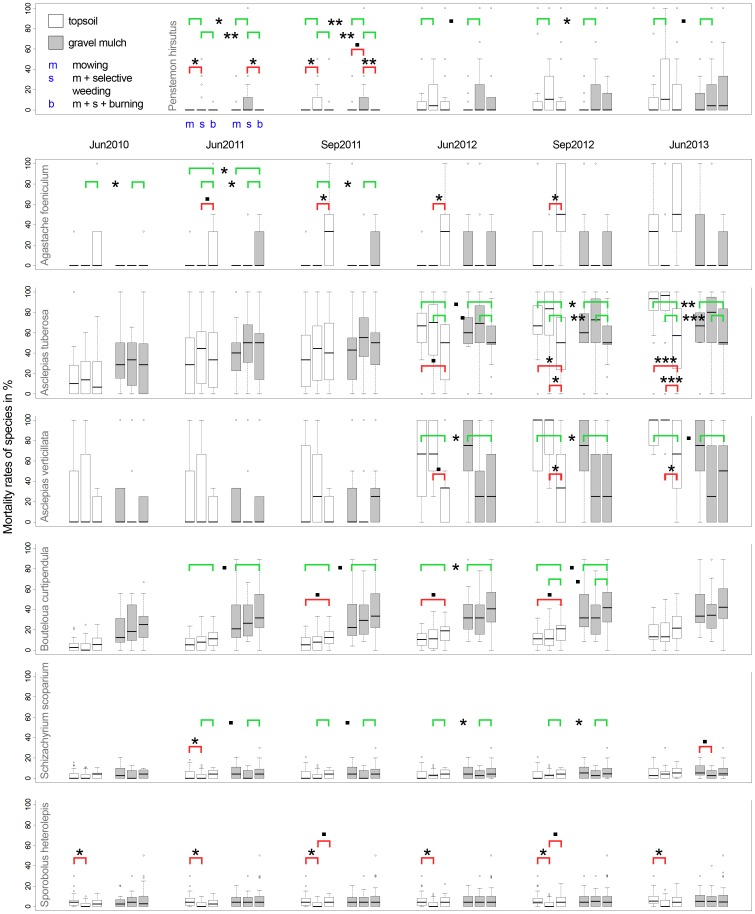
Effects of three different maintenance strategies [mowing (m), mowing + selective weeding (s), mowing + selective weeding + burning (b)] on mortality rates of planted target species between June 2010 and June 2013 differentiated by soil type. Significant differences between management types are indicated by red brackets and asterisks. P<0.1 * P<0.05 ** P<0.01 *** P<0.001. Asterisks on green brackets indicate significant differences between management types for the whole of the planting regardless of soil type. These results could not always be verified at the level of substrate units, but are mostly in accordance with the trend suggested by data distribution.

At the individual species level, seven species showed a significant response to management type: *Agastache foeniculum*, *Asclepias tuberosa*, *As. verticillata*, *Bouteloua curtipendula*, *Penstemon hirsutus, Schizachyrium scoparium* and *Sporobolus heterolepis* (see Fig. 1; for *Penstemon hirsutus* data assessment started in Jun 2011). Some of these species were heavily damaged by being burned, others seem to have profited from the treatment.

*Agastache foeniculum* had a tendency to higher average mortality rates under fire management (treatment b) during several assessments. When analysis was undertaken at the substrate unit level, those differences were found to be significant on topsoil sites only.

*Bouteloua curtipendula* had a general tendency to higher mortality rates on burned sites. At the substrate unit level, this was verified for topsoil plots only. Mortality rates of *Schizachyrium scoparium* were generally significantly higher under burning management compared to weeding management (treatment s). Analysis at the substrate level confirms this trend for both soil types, but mostly without verifiable significance. *Sporobolus heterolepis* mortality rates were lowest with weeding management and increased by additional burning on topsoil sites, whereas no differences were observed on gravel mulch sites.

Both *Asclepias* species had significantly lower average mortality rates after the second growing season on burned sites compared to sites managed by mowing and weeding or mow-only sites (treatment m). Significant differences between s-management and b-management were found in topsoil sites only. *Penstemon hirsutus* performed best on mow-only sites and regularly had significantly higher average mortality rates under weeding management on both types of substrate.

The remaining 25 species did not respond significantly differently to the different treatments.

Management type also did not have any measurable influence on target species total coverage values.

### Effect of maintenance type on target species vitality

Original sample sizes for detailed surveying of individual species ranged from 72 to 270, depending on the mixture-related frequency of each species. For a complete list of species, assessed criteria and sample sizes see table 2. Sample populations of *Asclepias verticillata*, *Dalea purpurea*, *Echinacea angustifolia*, *Liatris aspera*, *Penstemon ovatus* and *Ratibida columnifera var. pulcherrima* either died out or the numbers dropped below levels needed for statistical analysis. Significant differences in vitality criteria due to management treatment were found in three species: *Amorpha canescens*, *Monarda bradburiana* and *Schizachyrium scoparium*.

**Table 2:** Criteria for vitality assessment and sample size development of 14 prairie species.

Species	Data collection		orig. SS	Avg survival rate (%)			Assessment criteria							
	June	Sept.		in 2011	in 2012	in 2013	basal Ø	clump Ø	# of gen. shoots	# of veg. shoots	hgt of longest shoot	# of flowers	# of open flowers	# of flowers on longest shoot
Amorpha canescens		x	90	93.3	80.0	73.3			x	x				
Asclepias verticillata	x		144	80.5	46.5	38.2			x			x		
Bouteloua curtipendula		x	270	98.9	90.0	85.5	x		x					
Dalea purpurea	x		72	61.1	5.5	1.3			x				x	
Echinacea angustifolia	x		216	50.5	25.0	17.6			x				x	
Eryngium yuccifolium	x		164	94.2	85.9	80.5			x		x			
Liatris aspera	x		144	76.4	46.5	35.4			x					x
Monarda bradburiana	x		108	77.7	76.8	71.3		x	x					
Parthenium integrifolium	x		126	92.1	91.3	75.4	x		x					
Penstemon ovatus	x		144	62.5	37.5	25.0		x	x					
Ratibida columnifera var. pulcherrima	x		126	30.2	4.8	0.8			x				x	
Schizachyrium scoparium		x	270	100.0	99.6	97.8	x		x					
Solidago missouriensis		x	108	98.1	90.7	-	x		x					
Sporobolus heterolepis		x	270	99.3	99.3	98.5	x		x					

SS  =  sample size, Avg  =  average, gen.  =  generative, veg.  =  vegetative, hgt  =  height, length information values in cm.

*Amorpha canescens* produced significantly fewer vegetative shoots on burned plots regardless of soil type in 2011 and 2012 (significance levels varying between p<0.1. and p<0.01**). There was no measurable effect on the number of generative shoots. The original sample size was 90 individuals, of which 5 had died by the first assessment in September 2011. By the end of the study, 66 individuals remained, the majority of which grew on topsoil plots (56%).

*Monarda bradburiana* produced significantly more generative shoots on burned topsoil plots during all assessments (p<0.05* in 2011 and 2012, p<0.01** in 2013). No differences were observed on gravel mulched plots. Diameters of clumps were significantly larger on burned topsoil plots after 2011 (p<0.1. in 2012, p<0.01** in 2013). The original sample size was 108, which fell to 77 individuals towards the end of the study (44% growing on topsoil).

While basal diameters of *Schizachyrium scoparium* clumps were independent of management type, the number of flowering shoots was higher on burned topsoil plots during all years (p<0.05*). No effect was observed on gravel mulch sites.

### Effect of maintenance type on weed species presence and abundance and weed species community composition

Analysis of data on weed species presence and abundance clearly showed a significant influence of maintenance type. The greatest quantities of assessed weed species and the highest cover values were found on topsoil sites with m-maintenance, the lowest values were the result of s-management on gravel-mulched sites.

**Figure 2:**
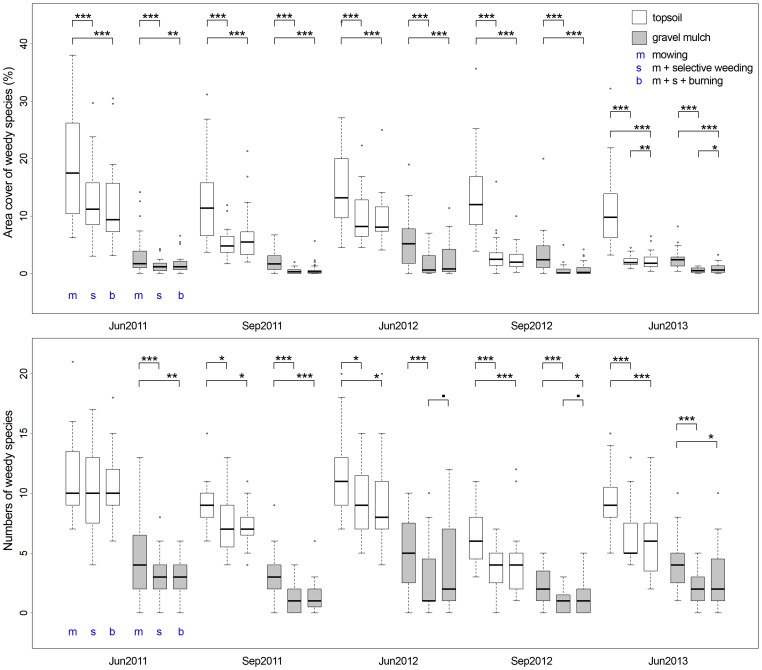
Effects of three different maintenance strategies on cover values and numbers of weedy species between June 2011 and June 2013 differentiated by soil type. Significant differences between management types are indicated by asterisks. P<0.1 * P<0.05 ** P<0.01 *** P<0.001.

Study plots that received m-treatment mostly had significantly higher weed species numbers and cover values than plots with s- or b-treatment (see Fig. 2), although the level of significance varied. Minor differences were found for the comparison of weed numbers of s- and b-treatments in 2012. Only the last assessment revealed significant differences between cover values of s- and b-treatments.

Differences in weed species community composition between m-plots vs. s-plots and b-plots were highly significant for all assessments (p<0.001***). The species that predominantly accounted for those differences generally included woody species (*Betula pendula*, *Clematis vitalba*, *Populus tremula*, *Salix aurita*, *S. caprea*) and herbaceous species of high growth and visual dominance (*Cirsium arvense*, *Conyza canadensis*, *Epilobium lamyi*, *Erigeron annuus*, *Solidago canadensis*, *Sonchus olearceus*).

Between s- and b-treatments, no significant differences in weed species community composition were found at any assessment.

### Comparison of labour input between different maintenance treatments

**Figure 3:**
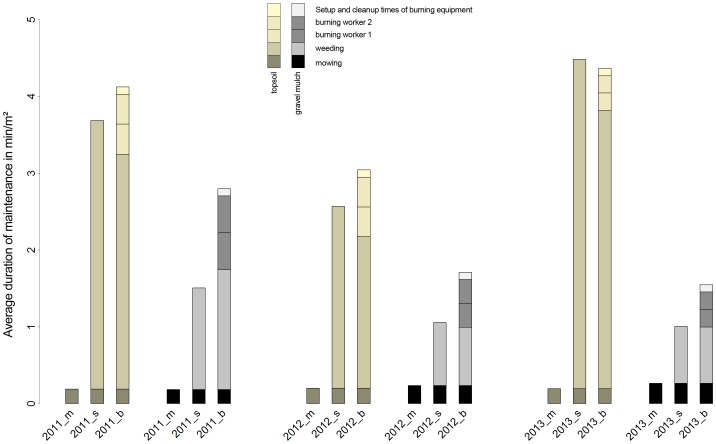
Maintenance times for mowing, selective weeding, and burning measures on the three different treatment sites (_m, _s, _b) and comparison of total maintenance duration for top soil and gravel mulch sites in 2011, 2012 and 2013.

On topsoil sites, total weeding times were highest for s-sites and lower on b-sites. On gravel mulch sites, weeding times on b-sites were mostly equal to or even higher than on s-sites. However, none of these differences were significant. Fig. 3 shows the total maintenance times for mowing, weeding and burning activities for the three maintenance strategies, separated by year and substrate. We included machine set-up and clean-up times (approximately 30 minutes per burning occasion) and increased personnel needed for burn control during the burning process (doubling of assessed burning times). We calculated this effort on the basis of one-third of the size of the total study site (equals 311 m^2^) and added these values in min/m^2^. We excluded fuel costs for the burner (depending on the runtimes), labour time needed for the acquisition and return of the burning equipment, fuel costs and operating costs of the transport vehicle and daily rental costs for the burning equipment from the analysis.

When all labour times related to burning activities were summed, the total duration of average maintenance on b-sites considerably exceeded s-site maintenance times, except in the case of topsoil sites in 2013, where total labour times were about equal.

## Discussion

### Effect of maintenance type on target species establishment

Only a few of the target species showed significant reactions to burning treatment in terms of mortality. Half of those species had higher average mortality rates; the other half had lower rates.

No obvious benefit of a fire management for the establishment was documented that would outweigh the increased costs of burning.

*Asclepias tuberosa* and *A. verticillata* had lower mortality rates on burned sites, especially on topsoil. Their rhizomes, which lie deep in the soil, protected them effectively from fire damage. No apparent reason accounts for the better performance on topsoil sites, though. *Agastache foeniculum* generally performed best on mowed but unburned sites. It is an early developing species with close-to-the-surface buds, which may have been significantly harmed by burning.

The higher mortality rates of both grass species on burned sites regardless of soil type were consistent for the duration of the study. It was clearly not a failure of initial establishment, but rather a continuous tendency to higher death rates under fire management that accounted for this.

At first glance, this appears to be inconsistent with current knowledge about the general facilitation of prairie grasses in natural prairies by dormant-season fire management, e.g. [33], [39], [43], [54]. None of these studies, however, evaluated the longevity of individual plants with regard to management, but instead they assessed the performance of an overall grass community by means of total productivity. It is not unlikely that substantially higher grass seedling rates on burned sites account for most of this facilitating effect [55], more than compensating for the shortened lifespan of the individual grasses under fire management. Since the horticultural use of prairie vegetation is not essentially different from other ornamental plantings in that they are focused on maintaining a distinct and fairly static design vision over the long term, species-specific reactions to alternative management regimes are critical and the documented fire effect was mostly counterproductive during this early development stage of the planting. It may become interesting again when the planting is fully developed and in need of corrective measures to preserve the original design idea.

One reason for the predominant similarity in mortality rates between b-sites and s-sites may have been the study design. We compared unburned and burned plantings, both of which were mowed and raked before the burning treatment. No standing biomass or litter remained, which would have created an organic mulch layer and consequently significant differences in site conditions, as would be found natural prairie communities (light-, temperature- and establishment barrier created by the remaining litter in unburned areas vs. clean-swept dark burned surface). The effect of burning therefore may have been strongly reduced owing to the similarities in the site conditions [56].

A second reason for the minimal differences between b-sites and s-sites may have been the development stage of the planting material at the beginning of the study. Because we planted rather than seeded the study site, the individuals had a sufficiently well-developed root system from the start, with at least eight weeks of the growing season left after the planting process. The first burning took place five months after planting. By that time, most individuals were well grown-in. The burning effect on mortality rates most likely would have been more distinct if we had included naturally rejuvenated specimens in the analysis, which are more easily damaged by fire. We would expect the same at later stages of development, when competition between densely growing mature individuals becomes an important factor for survival and reproductive success. At that stage, fire will most likely have a strong influence on established competitive hierarchies and may be used to steer the planting towards the long-term development objectives by adjusting burning dates during the season. Our data indicate that fire is more harmful than helpful for survival of the target species during the post-seedling stage but still early in the establishment phase of a planting development. In the context of designed prairie plantings in an urban setting, this is a very important aspect with regard to the pursued longevity and potential (self-) sustainability of a public prairie planting and needs to be investigated further. We are interested in moderately dynamic communities, where persistent plants sustain the original design picture for a long time, and necessary rejuvenation is also able to take place. If gaps are needed for the grasses to rejuvenate, then sustainable management of ornamental prairie plantings needs to provide this. Obviously, this need not necessarily be done by burning. It may also be achieved by raking mowed sites, thus avoiding the negative effects of burning on the mortality rates of stock plants.

### Effect of maintenance type on target species vitality

An influence of management type on species vitality was proven for only three species (*Amorpha canescens*, *Monarda bradburiana*, *Sch*i*zachyrium scoparium*). Burning increased basal diameters and numbers of flowers in two species. The third species developed fewer vegetative shoots, but the same number of generative shoots and therefore was not restricted in its reproductive potential. The overall effect of burning on vitality therefore was rated minimally positive, but negligible.

The general lack of a significant effect on the majority of the species (especially the grasses) was unexpected as common knowledge holds that burning has a favourable effect on the vigour and vitality of prairie species in general [57] and specifically on the vitality of prairie grass [33], [58], [59]. Again, the lack of significant effect was most likely caused by the similar preparations of s- and b-sites prior to the burning treatment. When standing biomass was removed by mowing and raking, no temporary nutrient surplus was available after burning to support more vigorous growth at the beginning of the growing season. Surface light conditions and soil surface temperatures after raking and subsequent burning were basically equal to those following a natural burning management, whereas nutrient availability was not. Temporary nutrient surplus is known to be an important factor in prairie species facilitation via burning, though [60], [61]. Since similar growing conditions on s-sites and b-sites yielded mostly equal results with respect to individual plant vitality, s-management was rated adequate, while b-management was rated dispensable.

### Effect of maintenance type on weed species presence and abundance and weed species community composition

The influence of management on the presence and abundance of weed species was highly significant. Omission of selective weeding measures resulted in increasingly weed-infested plots, lacking any visual quality of the original plantings, regardless of soil type. This rapid maldevelopment stood in contrast to the promoted low-maintenance of prairie plantings [18]–[21] during the establishment phase.

Differences between s- and b-plots were rare and of minor significance. We expected wintergreen species, especially wintergreen annuals, that were alien to the fire-adapted plant community to be significantly affected by the burning (as described by Hitchmough [62]), while perennial and geophytic species would be less affected. We either expected a substantial reduction in total weed coverage values or noticeable differences in the weed community composition; neither expectation was met.

A possible reason for this lack of difference may have been the early time of burning during the year. While native weedy vegetation was already in leaf, it was not fully developed and may not have been affected strongly enough by the burning treatment. We would expect a later date to be more effective (end of April, shortly before the prairie species shoots emerge). Where geophytes -an important component of public green spaces in Germany- are present, however, this might coincide with the emergence of their shoots or blooming. This needs to be considered when designing species mixtures.

A slight tendency to higher numbers and abundances of weeds on burned gravel mulch sites compared to unburned but weeded gravel mulch sites (see Fig. 2) was interpreted as a rather counterproductive effect of burning. This may have been due to a fertilising effect of the deposited ashes, which provided organic material and nutrients in gaps in the mulch and created a temporary nutrient surplus supporting germination. The mow-only treatment was abandoned in late summer of 2013 after the last data assessment due to an increasing *Calamagrostis epigejos* and *Cirsium arvense* infestation, which by June dominated most of the m-plots and started to influence neighbouring sites. Both species are known for their efficient vegetative spread as well as highly successful generative reproduction [63], [64]. Therefore, a regular s-management was introduced in order to relieve remaining target vegetation within the former m-plots and to prevent infestation of the well maintained s-plots and b-plots.

### Comparison of labour input between different maintenance treatments

While selective weeding proved to be essential for successful establishment and preservation of the original planting design, the additional expenditure of labour for burning had only a small influence on weeding times and planting development.

On topsoil sites, the average savings in weeding labour time on burned plots was generally smaller than the additional time investment for the burning treatment, resulting in higher total management times (Fig. 3).

On gravel mulch sites, the effect of burning on weeding times was only inconsistent and minor. Total maintenance times on burned gravel mulch plots were significantly higher than on s-sites.

On the whole, burning treatment significantly increased total maintenance times and costs, regardless of soil type, without any significant benefit for the plantings' development during the first three years of establishment.

## Conclusions

To estimate the general effect of different management techniques and especially the potential benefit of a burning treatment on prairie-like herbaceous mixed-plantings in public open space, several points were considered in this study.

We analysed the effect of fire on community and individual species mortality. At the community level, no effect of burning was registered. At the species level, only a few species reacted significantly, and those reactions were of an ambivalent nature: some species showed higher mortality rates under a burning regime (*Agastache foeniculum*, *Bouteloua curtipendula*, *Schizachyrium scoparium*), others seem to have profited from regular burning of the site (*Asclepias tuberosa*, *A. verticillata*, *Penstemon hirsutus*).

The comparison of different vitality criteria revealed no measurable effect of regular fire management on the majority of target species. We found significant differences in only three species, of which one was slightly affected by the burning (*Amorpha canescens*) and two thrived on it (*Monarda bradburiana*, *Schizachyrium scoparium*).

Analyses of species numbers and cover values of weedy plants revealed the critical impact of selective weeding activities on the establishment and maintenance of the target species populations compared to a mow-only treatment. No consistent differences were found between sites with selective weeding and weeding plus burning management, though. Neither the composition of weed species communities nor the numbers and abundances of weed species were significantly affected by the additional burning measures.

Summing up, mow-only treatment of mixed prairie plantings resulted in the loss of the original design in just a short time due to uncontrolled aggressive weed expansion, especially on topsoil sites. To evaluate the benefit of an additional burning treatment, the slightly increased vitality and lower average mortality of a small number of target species need to be weighed against higher average mortality rates of other target species and significantly increased input in labour and financial resources. Therefore, we conclude that there is no overall positive effect of burning on the establishment and maintenance of mixed prairie plantings on topsoil sites and even less on gravel-mulched sites. Considering maintenance costs, we conclude that the most effective management combination is mowing and raking in mid-spring plus regular selective weeding measures on gravel-mulched plantings. To evaluate the long-term effect of different management techniques on urban ornamental prairie plantings, rejuvenation success of target species and community dynamics need to be assessed in further studies.
